# Editorial: Modulating Glial Cells Phenotype: New Findings and Therapies

**DOI:** 10.3389/fnagi.2020.594870

**Published:** 2020-09-23

**Authors:** María José Bellini, Yolanda Diz-Chaves, Alberto Javier Ramos

**Affiliations:** ^1^Consejo Nacional de Investigaciones Científicas y Técnicas, Buenos Aires, Argentina; ^2^Laboratory of Endocrinology, The Biomedical Research Centre (CINBIO), University of Vigo, Vigo, Spain

**Keywords:** aging, inflammation, microglia, astroglia, therapies

Aging and neurodegenerative diseases are closely related, and the common point between both scenarios is neuroinflammation. Neuroinflammation is mainly mediated by glial cells, astrocytes, and microglia; while endothelial cells transfer the pro-inflammatory signals from periphery. The mechanism that underlies glial cells activation and neuroinflammation-related damage is not fully understood, being the study of glial cells during aging as well as neurodegenerative diseases, a key point to identify targets and to develop therapies that modulate adverse outcomes and mitigate neurodegeneration.

This Research Topic has produced a highly informative collection of original research and reviews, that cover multiple aspects for delving neuroinflammation and glial phenotypic changes in neurological diseases and aging. Researchers have presented their work and views to explain cellular and molecular mechanisms acquired by glial cells as well as possible interventions that can modify their functions and phenotypes associated with a broad spectrum of neurological diseases.

Serapide et al. started by focusing on the pivotal role of astrocytes activation in Parkinson's disease (PD), the most common age-dependent movement disorder. These authors tried a new approach to uncover the interaction between the astrocytes and neurons in PD mice brains. They demonstrate that grafting astrocytes alleviate oxidative stress in a parkinsonian brain by using MPTP-induced PD mice. Adding new information, Rabaneda-Lombarte et al. investigated the effect of MPP+ or rotenone to microglial/astroglial functions. Their manuscript provides information on the impact of PD-related toxins on the phenotype of glia, based on the evaluation of the mRNA levels of various gene products.

Deepen on astrocytes functions, the astrocyte-specific enzyme glutamine synthetase plays an essential role in supporting neurotransmission and in limiting ammonium toxicity. Moreover, deficits in Glutamine synthetase activity contribute to epilepsy and neurodegeneration. In this regard, Huyghe et al. extensively investigated the regulation of glutamine synthetase by PKA at T301 using biochemical and cell biological approaches. The authors successfully created two anti-phospho-GS antibodies and demonstrated that phosphorylation of T301 is critical for its activity in astrocytes. This modification results in decreased enzyme activity in a mouse model of epilepsy.

To provide new insights into microglial implication in behavioral changes induced by infections and mediated by pro-inflammatory cytokines, Savage et al. describe for the first time the effects of acute LPS on the ultrastructure of microglia in the mouse hippocampus. The ultrastructural changes found by the authors, provide an excellent starting point to determine pro-inflammatory and phagocytic pathways engaged by in sickness behavior.

Another interesting aspect of glial cells participation on pathological conditions was reviewed by Zang et al.. Their work was focused on the role of epigenetic regulation in the pathogenesis of glioma and discuss its value as therapeutic target for the treatment of this disease. Authors expand our knowledge on four epigenetic phenomena that occur in glioma: DNA methylation, abnormal microRNA, histone modifications, and chromatin remodeling. They review current epigenetic treatments for glioma, such as inhibitors of DNA methyltransferase and histone deacetylation inhibitors and argument the limitations of their applications due to the complicated pathogenesis of glioma.

Various articles in this collection focus on the role and mechanisms of astrocytes and microglia in the aging brain. Current issues and novel findings in the modulation of glial cells and the pathways able to promote healthy aging will provide a better understanding of cellular and molecular mechanisms toward a neuroprotective or a deleterious glial phenotype. Related with this topic, Matias et al. introduced the concept of astrocyte heterogeneity, which is a hot topic in the field. The review addressed diverse phenotypes of astrocytes with an emphasis on aging and neurodegenerative conditions. The authors discussed historical overview of astrocytes, astrocyte heterogeneity in healthy brain and pathological conditions, concept of “inflammaging,” and selective vulnerability. Also, they present the possibility of using glia as target for therapeutic strategies.

Expanding the knowledge of the changes undergone by astrocytes during aging, Zárate et al. investigated the role of Humanin, an astrocyte-release mitochondrial peptide, and the influence of hormonal milieu. They analyzed the role of this protein on synaptic dysfunction in the context of hormonal decline as a model of aging. The study of Humanin regulation by ovarian hormones in the brain is novel and highly interesting, given the plethora of molecular targets that these hormones have and that participate in mediating their neuroprotective and neuro-restorative.

Continuing with the study of glial-cells changes during aging, Falomir-Lockhart et al. investigated the effects of IGF-1 gene therapy on microglia. Importantly, these authors found that IGF1 therapy leads to a region-specific modification of aged microglia population. Microglia cells become dystrophic with aging; which contributes to basal central nervous system neuroinflammation being a risk factor for age-related neurodegenerative diseases. These authors' findings suggest that such a therapy may be useful to alter the function of dystrophic microglia effects.

Another study by Trias et al. explores the emergence of senescent microglia in spinal cord during paralysis progression in amyotrophic lateral sclerosis (ALS) rat model (SOD1G93A). The authors showed nuclear p16INK4a expression in activated microglia during paralysis progression as well as in a subpopulation of spinal motor neurons and astrocytes. They suggest that cellular senescence is closely associated with inflammation and motor neuron loss occurring after paralysis onset in SOD1G93A rats. The emergence of senescent cells could mediate key pathogenic mechanisms in ALS.

With the increment of life expectancy, preventive measures, and treatments for age-related cognitive decline, and dementia are of utmost importance. The work by Ano et al. shows that both chronic and acute administration of Iso-alpha-acids (IAA) prevents the neuroinflammation and cognitive decline associated with aging. The use of natural compounds as IAA, which are hop-derived bitter compounds in beer, represent an interesting therapeutic tool to ameliorate age-related negative outcomes.

Later on, Velasco-Estevez et al. used an aging transgenic rat model of Alzheimer Disease (AD) to study the expression of mechanosensing Piezo1 ion channels in amyloid plaque-reactive astrocytes. Piezo1 is a non-selective mechanosensory mediating cation channel. The authors found that aging and peripheral infection augment amyloid plaque-induced upregulation of mechanoresponsive ion channels, such as Piezo1, in astrocytes. Additional work is required to investigate the role of astrocytic Piezo1 in the Alzheimer's brain and its possible use as a novel drug target for age-related dementia.

Microglia polarization to an anti-inflammatory phenotype has been described to be neuroprotective. In the study by Jin et al., they linked microglial pro-inflammatory polarization to neuroinflammation and autophagy dysfunction, that are closely related to the development of neurodegeneration in Parkinson's disease. They demonstrate that TNF-α inhibits autophagy in microglia through AKT/mTOR signaling pathway, and autophagy enhancement can promote microglia polarization toward anti-inflammatory phenotype and inflammation resolution. This study adds information to establish which specific factors are responsible for disturbing the pro-/anti-inflammatory balance in areas where inflammatory stimuli induce neurodegeneration. Besides the study contributes to understanding the relationship between inflammation and autophagy processes.

Together these articles have brought several interesting understandings of neuroinflammation and glial cells activation in a range of neurological diseases and aging. We expect that this topic would expand our knowledge of the mechanisms whereby reactive glial cells could be detrimental or neuroprotective. Moreover, this comprehension could contribute for the development of therapeutics to limit neuroinflammation or even slow down the process of aging.

## Author Contributions

MB, YD-C, and AR contributed equally to this editorial, in its conception, writing, and revision. All authors approved the submitted version.

## Conflict of Interest

The authors declare that the research was conducted in the absence of any commercial or financial relationships that could be construed as a potential conflict of interest.

